# *“It was simply disturbing“ -* evaluation of the stress factors of nursing staff on special COVID-19 wards during the pandemic: a qualitative study

**DOI:** 10.1186/s12912-025-02773-y

**Published:** 2025-02-03

**Authors:** Lea Kiefer, Christian Volberg, Jan Adriaan Graw, Stefan Bösner

**Affiliations:** 1 Department of General Practice and Family Medicine, Philipps-University Marburg, Marburg, Germany; 2 Department of Anesthesiology and Intensive Care Medicine, Research Group Medical Ethics, Philipps University of Marburg, Baldingerstraße, 35043 Marburg, Germany; 3 Research Group Medical Ethics, Faculty of Medicine, Philipps University of Marburg, Marburg, Germany; 4 Department of Anesthesiology and Intensive Care Medicine, Ulm University Hospital, Ulm, Germany

**Keywords:** COVID-19, Nursing staff, Workload, Stress factors, Qualitative research, Moral distress

## Abstract

**Background:**

The COVID-19 pandemic has posed enormous challenges for healthcare systems worldwide. The strain on nursing staff working in special COVID-19 wards during the pandemic increased dramatically. To support nursing staff more effectively in similar situations in the future, it is important to identify specific stress factors to design effective support measures.

**Objective:**

To collect the experiences and lessons learned from nursing staff who have been affected by COVID-19 pandemic on a special COVID-19 ward that were perceived as stressful. The findings should contribute to the development of specific support measures for healthcare professionals.

**Design:**

Qualitative interview study.

**Setting:**

We interviewed 14 members of nursing staff who worked on COVID-19 wards at two University Hospitals about their working experiences during the pandemic.

**Results:**

We were able to identify 10 key stress factors. These included an increased workload, communication deficits, a difficult personnel situation, subjective pressure, the establishment of a new ward, a shortage of material resources, inadequate hygiene conditions, a lack of opportunities to cope with the situation, the absence of relatives and decision making.

**Conclusion:**

The identification of various stress factors highlights the urgent need for comprehensive support measures. These measures could include concepts for dealing with physical and psychosocial stress, the provision of resources and sufficient personnel support. It remains crucial to proactively take preventive and supportive measures to reduce the burden and moral distress of nursing staff and protect their health in the long term. Despite the pandemic, the implications of our findings remain relevant for the future.

**Supplementary Information:**

The online version contains supplementary material available at 10.1186/s12912-025-02773-y.

## Background

The COVID-19 pandemic has posed enormous challenges for the healthcare system worldwide. The rapidly increasing number of infections in Germany triggered concerns that the healthcare system would be overburdened by a possible shortage of nursing staff. As a result of the high number of patients, key resources in the healthcare system became scarce. This shortage affected not only material resources but also healthcare and nursing staff [1]. The state of emergency during the pandemic was accompanied by a high level of physical, mental and emotional stress. Many hospital and care facility staff members have reached the limits of their resilience, with potentially serious consequences [2, 3].

In May 2020, the World Health Organisation (WHO) warned of an increase in mental health symptoms and illnesses such as depression and anxiety. The impact of the pandemic on people’s mental health is worrying [4]. Mental stress also increased among the German population as a whole [5, 6]. In particular, however, care staff working with COVID patients were confronted with increased work-related stress and psychosocial strain [7]. The overworking of nursing staff resulted, among other things, in an increased tendency to leave the nursing profession [8].

It is crucial to develop measures to reduce the mental stress of care staff and strengthen their resilience. To develop appropriate measures to overcome these challenges, a thorough understanding of the specific stress factors faced by nursing staff is essential. This support is not only urgently needed in times of crisis such as a pandemic. The resilience of the healthcare system, particularly that of nursing staff, should also be strengthened regardless of the pandemic [9].

To provide adequate care for all COVID-19-positive patients with a serious development of symptoms and to protect other non-COVID-19 patients, special wards were set up for COVID-19 patients, hereinafter referred to as special COVID-19 wards. Patients with a COVID-19 infection from all departments were treated on these wards. Although these wards were not Intensive Care Units (ICU), some seriously ill COVID-19 patients in need of care were accommodated and treated there. If the progression was more severe or even life-threatening, which required more intensive monitoring and treatment, the patients were transferred to the Intensive Care Unit. While various studies have investigated the challenges of the pandemic for Intensive Care Units and nursing home staff, the aim of this study was to collect experiences in general and experiences perceived as stressful by nursing staff working in special COVID-19 wards during the pandemic [10, 11]. Our study aimed to contribute to the development of support measures for nursing staff by investigating the specific stress factors and their impact on the health of nursing staff.

## Methods

### Study design and setting

To uncover aspects that would not be considered in a quantitative survey, the use of a qualitative research design for data collection was considered appropriate [12]. The study was conducted at the University Hospitals of Marburg (Hesse) and Ulm (Baden-Wuerttemberg) in Germany. Both hospitals had specialised COVID-19 wards during the pandemic.

The ethics committees of the medical faculties of the University of Marburg (93/22) and the University of Ulm (131/23) authorised the conduct of the study and the study was registered in the German Clinical Trials Register (DRKS00030425).

The ‘Standards for Reporting Qualitative Research’ (SRQR) served as a reporting guideline, enabling a structured and transparent presentation of our results.

### Sample and study participants

We explicitly surveyed only nursing staff who were working on specialised COVID-19 wards during the COVID-19 pandemic. Most of the nursing staff interviewed worked on these COVID-19 wards during the first two waves of the pandemic, which lasted in Germany from March to May 2020 and from September 2020 to February 2021 [13]. All participants had to be at least 18 years old, have a good command of German language and give written consent to participate in the study. To ensure a representative sample and sufficient thematic saturation, the study participants were selected based on the “theoretical sampling” approach of grounded theory. Among other things, this means that data collection and data analysis run in parallel. As part of “theoretical sampling”, data collection is continued until theoretical saturation and contrasting is reached, at which point no more new information or insights are gained. At this point, the data collection process is concluded [14, 15]. Participants from different hospitals and wards, from different age groups and genders, with different years of professional experience and different professional qualifications (specialist nurses, nursing assistants, geriatric nurses) were interviewed.

### Recruitment

Recruitment was mainly carried out by the study team personally approaching potential study participants. If they showed interest in participating, we sent them information about the study in the form of a prepared information sheet. E-mails with information about the study were also sent to ward managers, and flyers with information and contact persons were distributed and posted in the clinics. In addition, we relied on the “snowball system” and asked the interviewees to motivate their colleagues to participate in an interview [16]. If potential interviewees allowed us to contact them, we called them by phone, answered questions and arranged an interview.

### Data collection and interviews

The data were collected from November 2022 to November 2023. Before the start, all participants received both written and verbal study information and provided written consent. After providing consent, the participants were asked to complete a form to collect sociodemographic data [16]. The interviews were then started. We conducted semi structured face-to-face interviews with nursing staff and asked them about stressful events and experiences that they associated with their work on the COVID-19 ward [17]. The questions and structure of the interview guide were discussed and finalised in advance by the Qualitative Research Working Group of the Institute of General Practice at the University of Marburg [16, 18]. The first interview served as a pilot interview. Afterwards, the interview guide and the form for recording the sociodemographic data were adapted again. As numerous relevant aspects had already been mentioned, the data from the pilot interview were integrated into the main analysis.

All interviews were conducted and recorded by Lea Kiefer and then transcribed verbatim using MAXQDA 2022 software and pseudonymized afterwards [19, 20]. At the time the interviews were conducted, Lea Kiefer had no personal or professional relationships with the nursing staff she interviewed.

### Data analysis

The data were analysed using qualitative content analysis according to Mayring [21, 22]. In view of the multilayered nature of the topic under investigation, qualitative content analysis offers a suitable methodology for the systematic and detailed evaluation and interpretation of the interviews conducted [23]. This allows new aspects and correlations to be identified to develop a comprehensive understanding of the stress factors mentioned and their effects on nursing staff.

In the first step of the analysis, the transcripts were read to develop an initial understanding of the experiences described. This was followed by the deductive definition of the main categories based on the interview guide. Furthermore, subcategories were formed within each main category by identifying the most important themes. From new aspects that were mentioned independently of the questions asked, we inductively created further categories and thus supplemented the category system until all interviews were fully coded [22, 24]. To ensure the validity and reliability of the project, we continuously reviewed our categories, discussed them with the research team (LK, SB, JG, CV) and made adjustments where necessary [22, 25].

## Results

### Study participants

A total of 14 participants were included in this qualitative study, 10 of whom worked at Marburg University Hospital (71.4%) and 4 at Ulm University Hospital (28.6%) in specialised COVID-19 wards during the pandemic. 12 of the respondents were female (85.7%), and 2 were male (14.3%). Among the study participants, 12 were qualified healthcare and nursing professionals, one was a healthcare and nursing assistant, and one was a geriatric nurse. There was wide variation in terms of age and professional experience. The age ranged from 24 to 65 years. Some participants had less than 5 years of experience, while others had over 20 years of professional experience. The interviews lasted between 18 and 64 min (mean 38.64; SD 5.31) and were conducted at the interviewee’s workplace. Table 1 contains a detailed list of the demographic characteristics of the nursing staff interviewed.

**Table 1:** Detailed characteristics of the nursing staff surveyed

Feature	Sample size
Participants; n	14
Workplace; n (%)	
Marburg University Hospital Ulm University Hospital	10 (71.4) 4 (28.6)
Gender; n (%)	
Female Male	12 (85.7) 2 (14.3)
Age; years (mean, SD)	24–65 (36,64 ± 12,54)
Vocational education; n (%)	
Nurse Nurse assistant Geriatric nurse	12 (85.7) 1 (7.1) 1 (7.1)
Professional experience, years; n (%)	
≤ 5 years 6–10 years > 10 years > 20 years	4 (28.6) 3 (21.4) 3 (21.4) 4 (28.6)
Working hours, %; n (%)	
100% > 50 - ≤ 80% ≤ 50%	10 (71.4) 2 (14.3) 2 (14.3)
Family status; n (%)	
Married/in a stable relationship Living alone	11 (78.6) 3 (21.4)
Work on the COVID ward; n (%)	
voluntary involuntarily not specified	10 (71.4) 3 (21.4) 1 (7.1)

### Main results

Through our surveys, we were able to identify 10 key aspects that were perceived by the surveyed nursing staff as significant stress factors during their work on the COVID-19 ward. Table 2 presents a summary of the 10 categories, including subcategories.

**Table 2:** Compilation of the 10 categories of stress factors, including subcategories

Categories	Subcategories
Increased workload	- Due to hygiene measures - Due to higher work volume - Due to many patients - Due to administrative tasks
Communication deficit	- Lack of communication between colleagues - Insufficient clarification of areas of responsibility & standards - Information deficit - Lack of experience with pandemic situation
Personnel situation	- Staff shortage - Unstable team structure & collaboration with (non-specialised) external colleagues - Lack of expertise
Subjective pressure	- Limited freedom of choice - Sense of obligation
Construction of new ward	- (Other) premises
Material scarcity of resources	-
Hygiene conditions	-
Lack of ability to cope with the situation	-
Absence of relatives	-
Decision making	-

In the following, the abbreviation I stands for Interviewer and the abbreviation P for Participant.

#### Increased workload

The surveyed nursing staff named the high workload during their work on the COVID-19 ward as an important stress factor.

*It was an extremely heavy workload. So*,* you hardly ever finished. (Interview 6)*

*I was in there for four or six hours without getting out. That was exhausting. (Interview 10)*

The high workload was mainly caused by a combination of several factors, including the burden of necessary hygiene measures, a greater amount of daily tasks, a larger number of patients and administrative tasks. These four factors also form subcategories in this category.

##### Due to hygiene measures

The first important factor is the need for hygiene measures. The interviews made it clear that these factors contributed significantly to the high workload and therefore to the overall workload. The nursing staff reported the challenge of having to wear isolation gowns for long periods of time in sometimes heated patient rooms.

**I**: *Did you also feel stressed here because of more work?*

**P**: *Extra work. That was a lot just the insulation clothing. It was a lot to carry the whole thing. In addition*,* what was also difficult was standing in the room all the time.… That made everything more difficult. (Interview 9)*

*We had to change beforehand and then just wear goggles*,* full visors*,* masks and double gloves. Then*,* it was sometimes VERY hot in the summer months. Yes. You had to put up with a whole shift. (Interview 11)*

Delays caused by the entry and exit of emergency cases led to further stress and time pressure.

*In addition*,* as soon as there was a monitor alarm*,* by the time you were fully inducted and were able to see the patient*,* much time had passed*,* which naturally caused even more stress. (Interview 12)*

##### Due to higher work volume

The increased workload also resulted from a greater work volume compared to that of the usual working day. This was mainly due to the serious state of health of the patients, who were increasingly dependent on care and support.

... *and then the people were truly ill. They were truly sick at the beginning. In addition*,* at the beginning*,* you had to do this and do that. (Interview 13)*

In particular, the challenges and difficulties associated with managing patients’ daily physical hygiene were emphasised.

*There was just so much washing to do.… Therefore*,* we always had to watch because we just did not manage to wash everyone every day. I always found that truly awful. (Interview 5)*

##### Due to the many patients

The third significant factor that contributed to the increased workload was the high number of patients. The interviewees reported a resulting reduction in patient contact during their work on the COVID-19 ward compared to their regular workplace.

*Simply because of the number of patients*,* you truly did not have that much time for the patients. You truly only had to look after them and then move on to the next one. That was not always so nice in any case. (Interview 5)*

*And then I was on late shift and from 2.30 p.m. onwards*,* the Z. (ward) was putting patients up every twenty minutes. We could not keep up at all. (Interview 12)*

They emphasised that this meant that there was only limited time available for individual patient care. It became the norm to only see patients sporadically once per shift, as opposed to interactions that usually occurred several times a day. This resulted in dissatisfaction, as they felt that they could not adequately care for the patients.

*However*,* there were also shifts where you only saw the patient once. That is just the way it was. (Interview 6)*

*So*,* this caring*,* which is what you actually like to do when you’re a nurse*,* fell by the wayside. In addition*,* I often felt sorry for him because I always had the feeling that he actually wanted to tell me more. However*,* I have to get out again because I only have so many minutes in the room because then I have to go on to the next one. This was also a bit of dissatisfaction. (Interview 7)*

One nurse even perceived the high number of patients as “mass processing”.

*Well*,* I think there were an awful lot of people.… Well*,* I thought it was kind of a mass processing. (Interview 13)*

##### Due to administrative tasks

The interviews revealed that a considerable amount of administrative work was part of the daily tasks of the nursing staff on the special COVID-19 ward. This workload exceeded the usual level, particularly in terms of time, and therefore posed a further challenge for the nursing staff.

*And the main person responsible was the ward telephone*,* where of course all the health authorities*,* relatives and so on had to be dealt with somehow… (Interview 1)*.

*Therefore*,* we spent a lot of time on all the paperwork. (Interview 4)*

#### Communication deficit

Based on the data analysis, a communication deficit was identified as a cause of nursing staff stress. We divided this category into the following three subcategories: lack of communication between colleagues, insufficient clarification of areas of responsibility and standards, and lack of information.

##### Lack of communication between colleagues

The interviews revealed that team communication suffered from many challenges on the COVID-19 ward, such as a lack of staff or a heavy workload. Instead of interacting as a team, the focus was on looking after new employees and caring for their patients. There was hardly any time for dialogue with other team members, which ultimately led to the team breaking up, as described by one nurse.

*However*,* you no longer truly communicated as a team*,* but*,* yes*,* each individual person had to train three or four people and somehow*,* yes*,* had to look after these people in addition*,* which truly broke things up. (Interview 1)*

##### Insufficient clarification of areas of responsibility and standards

According to the study participants, a further increase in stress resulted from the inadequate clarification of areas of responsibility and standards on the COVID-19 ward. The interviewees reported that they lacked clear work processes, for example, regarding the deisolation and transfer of recovered patients. The lack of clarity led to delays and ultimately to a shortage of beds and an increased workload.

*Yes*,* such fixed standards were missing. […] that you look at ok when can you actually deisolate them? That is why it often failed*,* that things like that just fell by the wayside. (Interview 1)*

It was also mentioned that the constant change of responsible doctors made the situation even more difficult. As with the nursing staff, doctors also changed very frequently. The reason for this could be that the COVID-19 wards were set up in addition to normal hospital operations. The newly established COVID-19 wards were not assigned to specific disciplines. Therefore, the doctors also came from all disciplines. In addition, there was a shortage of physicians. The search for the right contact person among the many and frequently changing doctors on the COVID-19 ward made the situation even more difficult for the nursing staff during the entire time on the COVID-19 wards.

*It felt like there was a different [doctor] every day and sometimes there were five or six doctors there. And each of them divided up an area with rooms and you didn’t even know who the contact person was. (Interview 4)*

##### Information deficit

It became clear that the study participants were burdened by a considerable lack of information while working on the COVID-19 ward. In particular, the lack of notification to nursing staff regarding the closure of their own ward and the transfer to the COVID-19 ward was highlighted. The sudden reorganisation caused some to feel overwhelmed and insecure.

*Came into work the next day*,* and our ward no longer existed. Therefore*,* X (ward) was closed. Then*,* we were told we were now the Covid ward. Yes*,* of course that makes you feel a lot. However*,* at first*,* you’re perplexed and you do not truly know “OK”. (Interview 3)*

*Back then*,* we did not know anything*,* and the next day*,* we were told*,* “You’re going over now. You’re going to be corona”. (Interview 13)*

The survey results also showed that the nursing staff felt inadequately instructed and prepared for their work on the COVID-19 ward. Medical staff reported that new colleagues in particular did not receive any instruction or training, which indicates poor organisation and coordination.

*We were not prepared. We did not even have an introduction on how to put on our infection control clothing properly. We were not told that. That only came later. (Interview 2)*

*And when I saw how the other colleagues came from the wards*,* they hadn’t been instructed at all.… When they saw how it went up here*,* they were like: “We were not told that at all.“. So as far as that was concerned*,* it was very badly organised*,* I’d say. (Interview 8)*

##### Lack of experience with the pandemic situation

The nursing staff interviewed described the challenges associated with their limited experience in dealing with the coronavirus and the associated pandemic situation, especially at the beginning of the pandemic. They felt that they did not know enough about the virus and its effects, which led to uncertainty and stress.

*A real fight without seeing my opponent.… I just did not know what I was fighting. It was not visible. (Interview 9)*

*However*,* you just did not know anything about it. So*,* I have to say… [I did not] feel quite so well protected at first. Because you just did not know how severe it was. (Interview 12)*

Overall, it became apparent that the communication deficit was particularly challenging while working on the COVID ward.

#### Personnel situation

We divided this category into the subcategories of staff shortage and unstable team structure and collaboration with (specialised) external colleagues.

##### Staff shortage

Our data analysis revealed that the prevailing staff shortage was another important factor on workplace stress. The interviewees described a high turnover of staff, which led to a constant search for replacements, causing the remaining staff to feel additional stress. This staff turnover was due in particular to the fact that only a few were willing to work on the COVID-19 ward or were able to cope with the stresses and strains there. Despite the challenges that the nursing staff faced while working on the COVID ward, some stated that they repeatedly volunteered to work there when necessary.

*Because many people were there for a day*,* they were overwhelmed and left. Another one less. The next day*,* we had to search again. (Interview 1)*

*However*,* I would have done it again*,* because we had an absolute personnel problem*,* that there were people doing it at all. (Interview 6)*

The study participants explained that they often did not have enough time to adequately care for the emotional and physical needs of patients due to the nursing staff crisis. Not being able to do justice to everyone was a burden for the nursing staff.

*And there was no time for these people because it was simply too much for the limited number of nursing staff who were there. (Interview 3)*

##### Unstable team structure and collaboration with (specialised) external colleagues

The study participants reported an unstable team structure, which was caused by frequent staff turnover and its consequences. According to the interviewees, this impaired cooperation within the team and favoured the development of a lone wolf mentality, which in turn made work more difficult and led to emotional stress.

*... everyone has become a lone fighter to a certain extent because the team was split up as a basic team… They no longer worked together… (Interview 1).*

*We had different pool staff every day*,* different people from the paediatric wards. So that was our core team*,* but there were also lots of others*,* and that was just difficult because you had to get involved with someone new every day. (Interview 4)*

Some of the interviewees were removed from their usual teams, were integrated into the COVID-19 ward team and had to familiarise themselves with a new team. They described this situation as challenging.

*I then joined a completely new team. We also had a few people from Intensive Care with us*,* paediatric nurses*,* who were of course truly nice*,* but it was truly overwhelming for me that I had to work with a completely new team*,* which I did not know at all…. (Interview 5)*

Some of the study participants continued to work in their regular team and familiar premises. This constellation proved to be a significant resilience factor, as this group reported fewer stress factors overall.

##### Lack of expertise

Some of the new team members had little professional experience and little specialist knowledge, as they often came from other disciplines, such as paediatric nursing. This placed an additional burden on the nursing staff, who had to familiarise themselves with the new staff members and at the same time provide increased care for them and their patients.

**I**: *Would you say it was an extra workload on the ward?*

**P**: *Yes*,* very much. Very*,* because there were always new and inexperienced nursing staff coming in. We had to teach them about hygiene and the medication that was administered. (Interview 11)*

#### Subjective pressure

Our data analysis showed that the study participants felt subjective pressure in connection with their work on the COVID-19 ward. A significant part of this pressure was caused by superiors in the workplace.

*Then*,* the pressure from above: “However*,* you have to*,* otherwise you will be distributed to all kinds of wards.“. (Interview 2)*

**I**: *Did you feel under pressure?*

**P**: *Extreme! YES. (Interview 3)*

Furthermore, the emotional pressure felt by the study participants became clear as they endeavoured to hide their stress from family members.

*Children sense that. In addition*,* you cannot fool them*,* and they realise: “Mum’s not well at the moment*,* and it is a lot for mum right now”. In addition*,* that puts you under even more pressure*,* because of course you do not want the children to determine. (Interview 3)*

There are two subcategories in this category, limited freedom of choice and sense of obligation.

##### Limited freedom of choice

The nursing staff interviewed described a limited right to self-determination in relation to the decision to volunteer to work on the COVID-19 ward. Some of them described how they were not actively asked about their willingness to volunteer. Instead, decisions were made by superiors, and then, only the established facts were communicated.

*Yes*,* I was at X (ward) at the time*,* and that is just the way it was. The X (ward) became corona*,* and then it was just like that. That was decided. (Interview 13)*

*Yes*,* so in the end you were not truly asked. (Interview 4)*

If they had a choice, a decision had to be made within a very short time, which led to an emotional burden for the interviewees and was perceived as regrettable.

*We were then informed that our team was going to the COVID ward as a whole but that there was an option for anyone who wanted to opt out and could not or did not want to be deployed elsewhere. To make this decision within hours or a very short time*,* in the end you were already in the situation and then you had to look retrospectively*,* do I want this or do I not want this. In addition*,* I did not truly like that. You started with an emotional burden straight away. (Interview 3)*

##### Sense of obligation

Another significant stress factor was the perceived sense of obligation. The interviewees emphasised that this feeling was particularly directed at the other team members and the patients. The nursing staff did not want to leave them alone. This intention of “not wanting to leave their team colleagues and patients in the lurch” (interview 3) was clearly emphasised in several interviews.

*Yes*,* it was voluntary in the sense that we did it because we simply did not want to leave our team alone. (Interview 2)*

*And despite everything*,* they simply did not want to leave their colleagues in the lurch*,* or the patients*,* because there was no one else who could have done it. (Interview 3)*

*You knew that if I decided from one day to the next that I could not do it anymore*,* I simply could not do it emotionally*,* and there was a situation where I sat in the car crying and thought I could not go there. (cries) However*,* you cannot do it either. So*,* you cannot drive there either. You cannot call an hour before the shift and say: “Here*,* I have just realised I’m panicking*,* I do not want to do this”. Then*,* your colleague is on their own. In addition*,* that is not what you want for yourself. That was truly bad. (Interview 3)*

It is clear that emotional stress has far-reaching effects on numerous areas. For example, our study revealed that nursing staff definitely felt emotionally overwhelmed and exhausted and felt the need to withdraw from work. Feelings of being overwhelmed, helplessness and emotionally shaken by the challenging situation were more pronounced by the Nursing Assistant and the Geriatric Nurse compared to the nursing staff. On the other hand, there was a feeling of responsibility towards their colleagues. The combination of these two aspects contributed to inner conflicts and illustrates the complexity of the perceived pressure and thus psychological stress that the nursing staff were confronted with during their work on the COVID-19 ward.

#### Setting up a new ward

The COVID-19 wards were not set up on existing and prepared premises but required the creation of new wards used specifically for this purpose. Setting up the wards within an extremely short timeframe meant a considerable amount of extra work for the medical staff. The nursing staff described this additional workload as a further stress factor in the workplace due to the time intensity and effort involved.

*There were only two of us.… We had to move an entire ward within a very short space of time. You cannot imagine what that meant. In total*,* the patient was unplanned*,* medication was cleared without help*,* and beds were moved. From the rubbish bins to the laundry bags.… It truly was a lot of work. (Interview 11)*

##### (Other) premises

One problem was that the rooms in which the new wards were set up were unfamiliar. The nursing staff described how this led to chaotic situations, particularly in emergencies. Furthermore, in other situations, considerable effort is required to assemble all the necessary materials, which significantly increases the time required on this ward compared to working on a normal ward.

*We did not know the premises*,* nor did we know where anything was. When an emergency situation arose*,* we had to search like a mad. So that was not truly nice. (Interview 2)*

The structural and infrastructural deficiencies of the rooms also posed challenges for the nursing staff. In particular, the room where dying patients were transferred to in order to be able to die peacefully seemed particularly problematic due to the structural defects.

*And it truly is an ancient ward. So*,* there’s no running water in the room*,* there’s no toilet*,* there’s nothing. Therefore*,* we had to pass the washing bowls through the sliding doors*,* the sliders through them*,* the measuring cups for the catheters*,* and everything.… In addition*,* we had a room for the dying where the ceiling fell down and water came out of the ceiling and where you could not lay anyone down. (Interview 4)*

#### Material scarcity of resources

The nursing staff interviewed cited the shortage of material resources as another key issue in relation to the stress experienced during their work on the COVID ward. In the interviews, the limited availability of basic medical equipment was identified as a stress factor.

*Yes*,* which of course put an additional strain on us: We had already discussed the supply of materials to some extent. It is also about the basics such as blood pressure cuffs*,* oxygen saturation sensors*,* oxygen masks and so on. Everything was missing at the back and front. (Interview 1)*

*And the worst thing I realised was that I had worked in a specialist lung clinic for seven years: We came to this new ward that had already been cleared. They had actually dismantled the oxygen lines. We had to go back and stock up on everything down here. (Interview 11)*

This shortage of basic materials made it difficult for medical staff to provide comprehensive care to patients. Adequate patient care had to be ensured with the few resources available, which in turn increased the stress and strain during the pandemic and work on the COVID-19 ward.

#### Hygiene conditions

The nursing staff criticised the inadequate hygiene conditions on the wards. In particular, the lack of adequate room to maintain hygiene standards was a major difficulty and was perceived as a cause for concern by the study participants.

*And it was very disastrous in terms of hygiene*,* because we truly only have*,* I think there were three sluice rooms or something like that at the back of the corridors*,* where hygiene could perhaps have been maintained. However*,* otherwise you’re simply out of this corona room*,*…. (Interview 5)*

They also noted the shortage of personal protective equipment, which was closely linked to the shortage of material resources and contributed to the lack of hygiene conditions and inadequate protection for nursing staff. They continued to feel insecure as a result.

*Then*,* there was a time when we did not have any masks. Or hardly any masks. Then*,* you had to think about which mask to put on when*,* how and where again. (Interview 2)*

*For example*,* we had an FFP2 mask for all rooms. Therefore*,* for ALL patients. As I said*,* we had to wear them repeatedly for all patients. (Interview 1)*

Furthermore, the disappointment at the lack of interest shown by employers in this context was clear.

*We had no airlocks for dressing and undressing. We had ten-centimetre slits under the patient room doors. Just open slits. We were not protected. In addition*,* the employer did not care. (Interview 3)*

#### Lack of ability to cope with the situation

The study participants predominantly perceived their time and work on the COVID-19 ward as stressful. We concluded that the lack of opportunity to cope with the situation, for example, through supervised dialogue sessions, was perceived by some of the interviewees as one of the key issues that contributed to the particular stress.

*We asked for supervision and did not obtain it. We said we all needed it. To work through things. However*,* nobody was interested. (Interview 2)*

In one interview, a nurse described how she wondered why no support, such as someone to talk to, was offered on the COVID-19 ward. Working on the ward was described as “disturbing” (Interview 3), and the nurse interviewed said that she often did not know how to deal with the situation. The fact that no one asked how the staff dealt with the situation was perceived as disappointing.

*I often actually laid in bed and thought: “Therefore*,* when some catastrophe happens*,* people get help. They get people to talk to; they have supervision*,* and there are ways to deal with it. In all possible ways”. In addition*,* that was not recognised there. No one even asked how we were dealing with it. It did not matter at all. In addition*,* it was simply disturbing. We did not even know how to deal with it. (Interview 3)*

#### Absence of relatives

The absence of relatives was a stress factor for the nursing staff. Some participants in the study emphasised that they lacked important help and support due to the absence of the patients´ relatives.

*On the one hand*,* it was an additional burden for us because the relatives were of course constantly calling. Second*,* the patients were completely isolated. So if you could at least see SOMEONE from the family*,* that would have been a great help. (Interview 2)*

*It would probably have made things a lot easier for us as well because many people also support us. (Interview 4)*

The overall situation between relatives and nursing staff became more tense due to the current ban on visits, which led to conflicts and arguments in which the staff was forced to set clear boundaries. Coping with this situation proved to be challenging.

*I could understand that the relatives became impatient and sometimes a bit snotty. However*,* it was more than usual. So*,* you had to argue more and say: “No*,* you cannot come in*,* that is the way it is now.” (Interview 7)*.

#### Decision making

The nursing staff described how, while working on the COVID-19 ward, decisions had to be made repeatedly based on the assessment of the prospects of success as to which patients could be given more comprehensive therapies and care and which conditions were futile. Some of them perceived that these decisions might also have been affected by limited resources. For the nursing staff, the frequency and burden of these end-of-life decisions was difficult and stressful and was associated with major emotional and ethical challenges.

*And clustering patients like “Here*,* we could still do something about that*,* we might be able to save something. However*,* here*,* it is hopeless anyway”*,* and you also had to make a decision for yourself.… Yes*,* that is just not nice. When someone died*,* it was just “Oh FINALLY a bed again”. (Interview 2)*

*And what I found worst of all*,* especially on X (ward)*,* was that we sorted the rooms afterwards according to how well they were doing and how badly. (Interview 4)*

## Discussion and conclusion

### Most important results

Our findings provide comprehensive insight into the challenges faced by nursing staff during their work on special COVID-19 wards and highlight the need to support them. Identifying stress factors is crucial to counteract the stress nursing staff may experience in future exceptional situations and to provide them with the right support.

Our results support the findings of other studies on the increase in the nursing workload during the pandemic [2, 10, 26–28]. This stress factor was caused by a high number of patients and an increased nursing workload due to the poor health of the patients [29]. Compliance with the prescribed hygiene measures, for example, wearing protective equipment, proved to be physically demanding and time-consuming and increased the workload [10, 11, 26]. A high level of bureaucracy, for example, reporting to health authorities, further increased the workload. Social and emotional aspects of the nursing profession took a back seat, which additionally worsened the overall situation [11, 30].

We identified communication deficits as stress factor. This finding correlates with the findings of other studies [26, 28, 31]. However, our results provide more details as we were able to determine that these communication deficits resulted from various aspects, such as a lack of communication, an information deficit and unclear regulations regarding responsibilities and standards.

The staffing situation on COVID-19 wards posed further challenges for nursing staff. Changes in team dynamics were not only observed in our study [2, 26]. Staff shortages, which not only prevailed during the pandemic but also continues to occur today, continue to pose a major challenge [32]. In other hospitals, this shortage led to extended working hours and placed an additional burden on nursing staff [11]. The remaining staff were confronted with an unstable team structure, which caused difficulties, particularly due to the integration of colleagues from other (specialised) fields [26]. A stable team was also identified as a factor for job satisfaction and performance in other studies, which may explain the challenges posed by the volatile team structure in our study [11, 33]. For future comparable situations, it would be advantageous if teams were deployed in their familiar working environment and with colleagues with whom they are already familiar.

Subjective pressure proved to be another significant stress factor in our survey. Some nursing staff felt that it was their responsibility to work on the special COVID-19 ward and thus contribute to the fight against the pandemic. This is confirmed by the results of other studies [28, 34]. The nursing staff interviewed described a limited right to self-determination in relation to the decision to volunteer to work on COVID-19 wards. Previous research has shown that pressure from superiors can have a stressful effect [35]. There was also pressure not to let the team down, which is comparable to findings of increased camaraderie and morale in other job situations [26].

The physical environment can act as a stressor and has a negative impact on nursing staff [27, 36]. This was noticeable in the spatial reorganisation of the COVID-19 wards at the Marburg and Ulm sites. This led to considerable extra work and additional stress for the interviewees, who were also unfamiliar with the new premises.

The shortage of materials, particularly in terms of personal protective equipment, care utensils and suitable premises, further exacerbated the situation. Similar experiences were also reported from other locations [28, 37]. A shortage of protective equipment can lead to discomfort. The nursing staff were also concerned about their own health and safety as a result of the poorer hygienic conditions. The findings emphasise the urgent need for sufficient protective equipment and compliance with prescribed hygiene regulations to ensure the health of nursing staff.

Due to the lack of visiting options as a result of the lockdown the nursing staff no longer received significant support and relief [11, 38]. Conflicts and disputes even made relatives an additional burden at times [39]. While working on the COVID-19 wards, the nursing staff were confronted with challenging and stressful situations, particularly with regard to the treatment of the patients. For some of them, this burden was severe and led to moral and psychological trauma [26, 40, 41]. Nursing staff who have faced the challenges of working on a COVID-19 ward should receive appropriate psychosocial support [40]. Some respondents also expressed a desire for such professional support to cope with their stress and were disappointed that this request was not honoured. It became clear that the various stress factors partly cause and influence each other in a complex interplay. This in turn can lead to an increase in the feeling of stress and can result in moral distress for nursing staff, which underlines the importance and significance of close support measures [42, 43]. Moral distress can be described as a situation in which one recognises that one should do the right thing, but institutional guidelines and circumstances make it almost impossible to act accordingly [44]. Our findings and the statements of the study participants reflect precisely this definition. Furthermore, our findings on the causes of moral distress are consistent with the findings of other studies. These also identified conflicts in professional relationships, not being able to care for patients as one would like, lack of information, resource scarcity, staff shortages and organisational structures as causes, to name just a few consistent aspects [45, 46]. Moral distress is one of the reasons why nursing staff are reluctant to come to work, reduce their working hours or leave the healthcare sector altogether [46, 47]. Proactive preventive and supportive measures should be taken to protect the mental health of nursing staff and prevent the development of moral distress and, in extreme cases, the loss of them [11, 41, 48–50]. Possible solutions could include, for example, the creation of a support system in hospitals that helps nursing staff in such situations to deal with the emergency by providing them with someone to talk to. In addition, training could be introduced that addresses how to deal with moral distress [45, 47].

### Strengths and limitations

Our study made it possible to gain in-depth insights into the perspectives and experiences of nursing staff. Given the complexity of the topic, the use of a qualitative research design was considered appropriate to shed light on aspects that might not have been captured in quantitative surveys. We decided to conduct semi-structured guided interviews. This methodology enabled the nursing staff interviewed to freely describe their experiences and perspectives. However, this approach also poses methodological challenges. In particular, it makes it more difficult to differentiate exactly whether the statements described were actually uttered verbatim and the situations described took place in the form described or were merely perceived as such. Our data analysis was both deductive and inductive. This allowed us to identify stress factors that may not have been explicitly covered by the interview guide.

Some study participants mentioned only a few stress factors, which made it difficult to conduct the interviews, as the guidelines were not designed to address perceptions of little or no stress. One reason for the low stress could be that these interviewees all came from a team that was constant and worked on familiar premises [33, 36].

Another limitation of our study was recruitment. Although we endeavoured to compile a broad and representative sample, the motivation to participate in the study among the nursing staff was limited. This also extended the recruitment period. The results are based on the experience reports of 14 nursing staff members from Ulm and Marburg. Expanding the sample to include more hospitals, regions and a larger number of participants could make the results more generalizable. The predominantly female sample reflects the gender distribution in the healthcare sector.

The continuous exchange within our research team and our qualitative research group about the methodology used and the data collected allowed us to minimise subjective interpretations and strengthen the validity of the results.

### Conclusion and implications for practice

The COVID-19 pandemic posed major challenges for nursing staff working on special COVID-19 wards and led to increased work-related stress and psychosocial strain on nursing staff worldwide. This stress can have serious consequences, such as mental illness. The identification of various stress factors, including increased workload, communication deficits, staff shortages, resource scarcity and ethical dilemmas, underlines the complexity of the situation and the urgent need for comprehensive support measures for medical staff in times of crisis such as the COVID-19 pandemic. These measures could include sustainable concepts for dealing with physical and psychosocial stress, adequate provision of resources such as personal protective equipment and sufficient staff support. Efforts should be made to avoid moral distress, which is one of the main reasons for medical staff on leaving the healthcare sector.

Despite the pandemic, the implications of our findings remain relevant for the design of future working conditions and the development of support measures for nursing staff. It remains crucial to proactively take preventive and supportive measures to reduce the burden on nursing staff and protect their health in the long term.

## Electronic supplementary material

Below is the link to the electronic supplementary material.

Supplementary Material 1

## Data Availability

To protect the privacy of the study participants, no original data could be published. The data supporting the results of this study and additional material are available upon request from the authors.
