# Supplementary material for: “It was simply disturbing“ - evaluation of the stress factors of nursing staff on special COVID-19 wards during the pandemic: a qualitative study
Source: BMC Nurs. 2025 Feb 3;24:120. doi: 10.1186/s12912-025-02773-y (PMC11789282; doi:10.1186/s12912-025-02773-y)
Supplement: Supplementary file 1 — Supplementary Material 1 [file 12912_2025_2773_MOESM1_ESM.docx]

Interview guide

Introduction: Thank you for agreeing to be interviewed. We are interested in finding out if there were any events during your work on the Covid ward that affected you and if you are still feeling any effects. This is your personal opinion and there are no 'right' or 'wrong' answers.

The interview will take about 60 minutes. However, you can stop the interview at any time to take a break or end it. I have brought enough time with me and will organise it to suit you. Please don't hesitate to let me know if it gets too much for you.

I will record our conversation with this digital device so that we can analyse the results scientifically afterwards. The recording will be transcribed and pseudonymised so that it cannot be directly linked to your name or personal details. Do you agree to this? I will ask you again after the interview if you still agree.

Do you have any questions? Then let's get started.

**Questions:**

You worked on a special Covid ward during the Covid pandemic. Would you like to start by telling me about your time and work there?

How would you describe your work on the ward during the pandemic compared to before?

What thoughts and feelings did you have when you went to work?

- Additional: Theme: *Anxiety:* Did anxiety play a role? If so, what were or are you afraid of?

If you felt an extra burden, how did this make itself felt?

Were there any situations that were particularly stressful for you or that you had to think about for a long time?

- Additional: *Moral Burden Issue:* Have you ever had to make decisions that felt morally wrong?

Have you experienced stress while working on the Covid ward?

- Additional: If stress levels increased: How has this affected your personal wellbeing?

What changes have there been in your contact with patients? And to what extent did you find these stressful?

- Additional: *Relatives:* What role did the absence of relatives play?

Did you feel more confronted with death? If so, what was it like for you?

Have you noticed any changes in your private life as a result of your work on the Covid ward?

- Additional: *hobby/balance, support:* reduction of stress, thoughts about work, friends turning away, etc.

How has the pandemic situation affected your cooperation with colleagues?

Have you personally noticed any changes in the working situation on the ward as a result of the Covid vaccination? If so, what has changed?

Nursing and the pandemic have often been in the public eye in recent months. How has this public discussion about nurses and the pandemic affected you?

Could you describe any positive aspects of the last few months or would you say there have been none?

- Additional*: Appreciation Issue:* Has there been anything that has made you feel appreciated? Or something that made you feel the opposite?

Conclusion: Finally, do you have any suggestions / criticisms / other issues you would like to raise?

Are you still happy for this recording to be used for our scientific analysis?

Thank you for your time!

If the interview is cancelled

May the data already collected be used?

o YES

o NO

____________________________ ___________________________________

Place, Date Signature
